# Pathophysiology of GPCR Homo- and Heterodimerization: Special Emphasis on Somatostatin Receptors

**DOI:** 10.3390/ph5050417

**Published:** 2012-04-27

**Authors:** Rishi K. Somvanshi, Ujendra Kumar

**Affiliations:** Faculty of Pharmaceutical Sciences, Division of Pharmacology and Toxicology, The University of British Columbia, Vancouver, BC, V6T 1Z3, Canada

**Keywords:** somatostatin, somatostatin receptors, heterodimerization, G proteins, GPCRs, Pb-FRET

## Abstract

G-protein coupled receptors (GPCRs) are cell surface proteins responsible for translating >80% of extracellular reception to intracellular signals. The extracellular information in the form of neurotransmitters, peptides, ions, odorants etc is converted to intracellular signals via a wide variety of effector molecules activating distinct downstream signaling pathways. All GPCRs share common structural features including an extracellular *N*-terminal, seven-transmembrane domains (TMs) linked by extracellular/intracellular loops and the *C*-terminal tail. Recent studies have shown that most GPCRs function as dimers (homo- and/or heterodimers) or even higher order of oligomers. Protein-protein interaction among GPCRs and other receptor proteins play a critical role in the modulation of receptor pharmacology and functions. Although ~50% of the current drugs available in the market target GPCRs, still many GPCRs remain unexplored as potential therapeutic targets, opening immense possibility to discover the role of GPCRs in pathophysiological conditions. This review explores the existing information and future possibilities of GPCRs as tools in clinical pharmacology and is specifically focused for the role of somatostatin receptors (SSTRs) in pathophysiology of diseases and as the potential candidate for drug discovery.

## Abbreviations

AR: adrenoceptor
cAMP: cyclic adenosine monophosphate
AT1R: Angiotensin receptor 1
Ang II: angiotensin II
CNS: Central nervous system
*C*-tail: Carboxyl Terminal Tail
CXCR: Chemokine receptor
CO-IP: Co-immunoprecipitation
DR: Dopamine Receptor
ECL: Extracellular loop
EP1: prostaglandin E1 receptor
ER: Endoplasmic reticulum
ERKs: extracellular signal-regulated kinases
EGFR: Epidermal growth factor receptor
FSHR: Follicle-stimulating hormone receptor
FSK: forskolin
GABAR: gamma-aminobutyric acid receptor
GPCRs: G-protein coupled receptors
GRK: GPCR kinases
hSSTR: human somatostatin receptor
HA: hemagglutinin
HEK-293: human embryonic kidney-293
ICL: Intracellular loop
MAPK: mitogen-activated protein kinase
MR: muscarinic receptor
NMDAR: *N*-Methyl-D-aspartate receptor
ORs: Opioid Receptors
Pb-FRET: Photobleaching-fluorescence resonance energy transfer
PKC: Protein kinase C
PTX: pertussis toxin
RTK: Receptor tyrosine kinase
SSTRs: somatostatin receptors
TRs: Taste Receptors, V2R, Vasopressin receptor 2, 5-HT, Serotonin Receptor
CaSR: Calcium Sensing receptor
FCS: Fluorescence correlation spectroscopy
PD: Parkinson’s Disease

## 1. Introduction

G-protein coupled receptors (GPCRs) are the most prominent cell membrane receptor proteins which function in conjunction with second messengers and are involved in the regulation of many downstream signaling pathways. Several pathological conditions have been associated with the abnormal function or imbalance in GPCRs expression and have drawn great attention of pharmaceutical industries as an initiative to develop new drugs targeting several diseases like cancer, neurodegeneration, diabetes, pain and inflammation. It is therefore not surprising that some 50% of drugs currently on the market are associated with GPCRs and account for >40 billion USD annual revenue [1]. As early as 1993, Attwood and Findlay made an unique effort to classify GPCRs on the basis of the sequence of seven hydrophobic domains [2]. In 1994, Kolakowski presented the A–F classification system for all receptors which bind to G-proteins and the remaining were assigned to the O (other) family [3]. Bockaert and Pin in 1999 used structural and ligand binding criteria to segregate GPCRs into family 1–5 [4]. The most comprehensive classification of GPCRs was accomplished on the basis of phylogenetic criteria by Fredriksson and colleagues [5,6]. Today, >800 GPCRs exist in the human genome and have been classified in five distinct families. Amongst all classes of GPCRs, the rhodopsin family is the largest and most studied, accounting for more than 650 receptor types, whereas the remaining four classes of GPCRs have relatively less number of receptors, ranging from 15–36 [7].

The functional entity of GPCRs has been controversial and it was believed that the majority of GPCRs at cell surface exists and functions as monomers. However, in last ten years significant progress has been made and now it is widely accepted that most if not all GPCRs function as homodimers and/or heterodimers within the family or with members of other receptor families. The process of receptors dimerization can occur as early as during protein synthesis and its sorting at the level of endoplasmic reticulum (ER) [8,9]. As described earlier, vasopressin, oxytocin and calcium sensing receptors (CaSR) exist as dimers in the ER [10,11]. With the recent advances in methodology to ascertain protein-protein interactions, it has become clear that dimerization is not a unique property of GPCRs and it has also been expanded to many other receptor subtypes, including ligand gated channels such as *N*-Methyl-D-aspartate receptors (NMDARs) and the members of the receptor tyrosine kinase family [12,13]. Members of the receptor tyrosine kinase family such as epidermal growth factor receptors (EGFRs), commonly known as ErbBs, have been studied extensively for homo- and/or heterodimerization with significant pathological importance, specifically in different type of tumors [14]. EGFR in basal state exist as monomers at the cell surface and exhibit homo- and/or heterodimerization in the presence of ligands or even in case of over-expression. Recent studies have shown that EGFR heterodimerization is not restricted within the family, but also constitute a receptor complex with the members of GPCR family, such as somatostatin receptors (SSTRs) [15,16,17].

To study receptor dimerization different methods have been employed, including classical methods such as co-immunoprecipitation (Co-IP) to advance fluorescence based biophysical techniques, which exploit epitope tags of the receptors when expressed in heterologous system [18] (Table 1). As many GPCRs are expressed endogenously, HEK-293, CHO-k1 and COS cells have been instrumental for the characterization of GPCRs [19]. The use of recombinant DNA expression systems has proved to be an influential tool for this objective as various chimeric and mutant GPCRs are constructed [20]. These chimeric molecules were used recently to delineate the functional properties of the receptors in heterologous systems. Like for complementation assay, signaling deficient SSTR5 when expressed with binding deficient SSTR5, restoration of receptor signaling and functionality was observed [21]. Similarly, two non-functional GPCR chimeras, one with first five transmembrane domains (TMs) of the α_2_-adrenoceptor (α_2_AR) and last two TMs of muscarinic receptor 3 or *vice versa*, displayed restoration of receptor functionality [22]. Similar results were also demonstrated for the angiotensin 2 receptor, luteinzing hormone and vasopressin receptors (V2R) [23,24,25,26,27]. Most convincing evidence for the functional significance of GPCRs was shown for β-adrenoceptors (β-ARs), as a peptide mimicking TM VI of the β_2_AR resulted in the blockade of dimerization and agonist induced signaling [28]. These observations have served as a platform to delineate the structure and function studies and further characterization of many other GPCRs [28]. The modulation of pharmacological properties including binding properties, receptor trafficking and signaling of receptors upon dimerization has been observed in many studies involving opioid receptors (ORs), ARs, SSTRs and Dopamine Receptors (DRs). The existence of monomeric and oligomeric receptors on the cell surface has always been a point of debate. Many of the drugs targeting GPCRs were based on the monomeric form of the receptors however, the ratio of proportional distribution of monomeric/homodimeric/heterodimeric forms of the receptors existing on the cell surface is largely elusive [29].

SSTR subtypes are members of the GPCRs super family and belong to Class A Rhodopsin type subgroup. Many studies emerging from authors’ lab and elsewhere have demonstrated SSTR subtypes ability to form both homo- and/or heterodimers with members of the same or distantly related receptor subtypes [15,16,21,30,31,32,33,34,35,36,37,38,39,40]. In the present review, we highlight the functional significance of G-proteins and key receptor structural motifs which are responsible for receptor signaling and dimerization. However, this review is focused around the member of Class A GPCR namely SSTR subtypes and their interaction with DRs, ORs and ARs as well as RTKs. Furthermore, we also discuss the functional significance of this interaction with possible clinical implication in the process of drug discovery.

**Table 1:** The list of techniques used to determine homo- and/or heterodimerization.

Method	Receptors	References
**Complementation Assay**	Somatostatin receptors 1, 4 and 5	[21,36]
	Calcium sensing receptor	[41]
	Muscarinic M2 and M3 receptors	[42]
	Muscarinic M3/α2 Adrenoceptor	[22]
	Dopamine receptor 2/3	[43]
	GABAR_1_/GABAR_2_	[44]
**Co-Immunoprecipitation**	α,β-Adrenoceptors	[45,46]
	Dopamine receptors	[47,48]
	Opioid receptors	[49,50]
	Chemokine receptor 2	[51]
	Somatostatin receptors	[31,32,34,35,36,52]
	Somatostatin receptor 2/µ-Opioid receptor	[53]
	β-Adrenoceptors/Somatostatin receptor 5	[37,40]
	Somatostatin receptor 2A/3	[52]
	Angiotensin receptor 1/Cannabinoid receptor1	[54]
	β_2_ Adrenoceptor/Opioid receptor	[55]
	GABAR_1_/GABAR_2_	[56,57]
	Calcium sensing receptor/Glutamate Receptors	[58]
**FRET**	α-Adrenoceptors	[45]
	Thyrotropin receptor	[59]
	Neuropeptide Y receptor	[60]
	Dopamine receptor 2	[61]
	Chemokine receptor 2 and 5	[62]
	Somatostatin Receptors	[63,64]
**Photobleaching-FRET**	Somatostatin receptors	[21,31,32,34,36,38]
	Gonadotrophin-releasing hormone receptors	[65,66]
	Somatostatin receptors/Dopamine receptor	[30,35]
	Somatostatin receptor 5/β-Adrenoceptors	[37,40]
	Somatostatin receptor 4/µ-Opioid receptor	[67]
	Somatostatin receptor 4/δ-Opioid receptor	[39]
	Somatostatin receptors/EGFRs	[15,16,17]
**BRET**	β-Adrenoceptors	[68]
	Thyrotropin-releasing hormone receptor	[69]
	Opioid receptors	[70]
	Chemokine receptor 4 and 5	[71]
	Adenosine receptor	[72,73]
	Oxytocin Receptor	[10]
	Vasopressin Receptor	[10]
	Adenosine 2a receptor/Dopamine receptor 2	[73]
	Adenosine 2a receptor/Purinergic receptor 2	[72]
	Oxytocin/Vasopressin receptors	[10]
	Angiotensin receptor 1/Cannabinoid receptor 1	[54]
**TR-FRET**	δ-Opioid receptor	[74]
	β_2_ Adrenoceptor/δ Opioid receptors	[74]
	Histamine 4 receptor	[75]
	GABAR_1_/GABAR_2_	[76]

## 2. G-Proteins are Powerful Regulator of GPCR Signaling

As discussed above, GPCRs respond to distinct ligands and exert an important morphological, biochemical, physiological and pathological role in a receptor selective manner. Upon activation, GPCRs couple to heterotrimeric G-proteins resulting in the dissociation of a heterotrimeric complex of G_αβγ_ into G_α_ subunit and G_βγ_, which in turn activates downstream signaling via different isoforms of adenylyl cyclase (AC) [77,78,79,80]. Studies have shown the vast diversity following interaction between GPCRs and G proteins in activation of various signaling pathways [79]. Heterotrimeric G-proteins are generally classified into four families based upon the similarity in amino acid sequences: G_s_, G_i_, G_q_ and G_12_[81]. A concise description of some of the GPCRs coupling with distinct G proteins (G_i_, G_s_ and G_q_) is described in Table 2.

**Table 2:** Examples of GPCRs coupling to certain G Protein subclasses (G_i_, G_s_ and G_q_).

G Protein	Receptor Subtypes	References
**G_i_ protein**	Chemokine Receptor	[82]
	Opioid Receptor	[83]
	Somatostatin Receptor	[84]
	Neuropeptide Y Receptor	[85]
	Melatonin Receptor	[86]
	Cannabinoid Receptor	[87]
	Sphingosine-1-phosphate Receptor	[88]
	Histamine Receptor	[87]
	5-hydroxytryptamine	[89]
	Dopamine Receptor	[87]
	Muscarinic Receptor	[90,91]
	Formyl-methionyl peptide Receptor	[92]
**G_s_ protein**	Vasopressin receptor 2	[87]
	Adrenoceptors	[93]
	Prostaglandin E receptor subtypes	[94]
	5-hydroxytryptamine receptor subtypes	[89]
	Melanocyte-stimulating hormone receptor	[95]
	Melanocortin receptor subtypes	[96]
	Relaxin receptor subtypes	[97]
	Adenosine receptor	[98]
**G_q_ protein**	Vasopressin receptor subtypes (V1a and V1b)	[87,99]
	Muscarinic acetylcholine receptor subtypes	[90]
	Gonadotropin-releasing hormone receptor	[100]
	P2Y purinoceptor subtypes	[101,102]
	Bradykinin receptor subtypes	[103]
	Oxytocin receptors subtypes	[99]
	Gastrin/cholecystokinin type B receptor	[104]
	Neuromedin U Receptor subtypes	[105]
	Neurotensin Receptor	[106]
**G_i/s_ proteins**	Glycoprotein hormone receptors	[107]
	β-Adrenoceptors	[108,109]
**G_i/q_ proteins**	Platelet activating factor receptor	[87]
	Sphingolipid (S1P3)/Lysophospholipid receptor (LPA2)	[81]
	Galanin receptor 2	[110]
	Endothelin B	[111]
**G_q/s_ proteins**	Calcitonin Receptor	[112]
	Parathyroid hormone receptor	[113]
	Cholecystokinin-Areceptor	[87]
**G_q/i/s_ proteins**	Prostaglandin E3 receptor	[94]
	Thyrotropin receptor	[107]
	Luteinizing hormone receptor	[87]
	Lysophospholipid receptor subtypes	[114,115]

Data presented in this table has been generated from the following references [81,87,116,117,118].

**Figure 1:**
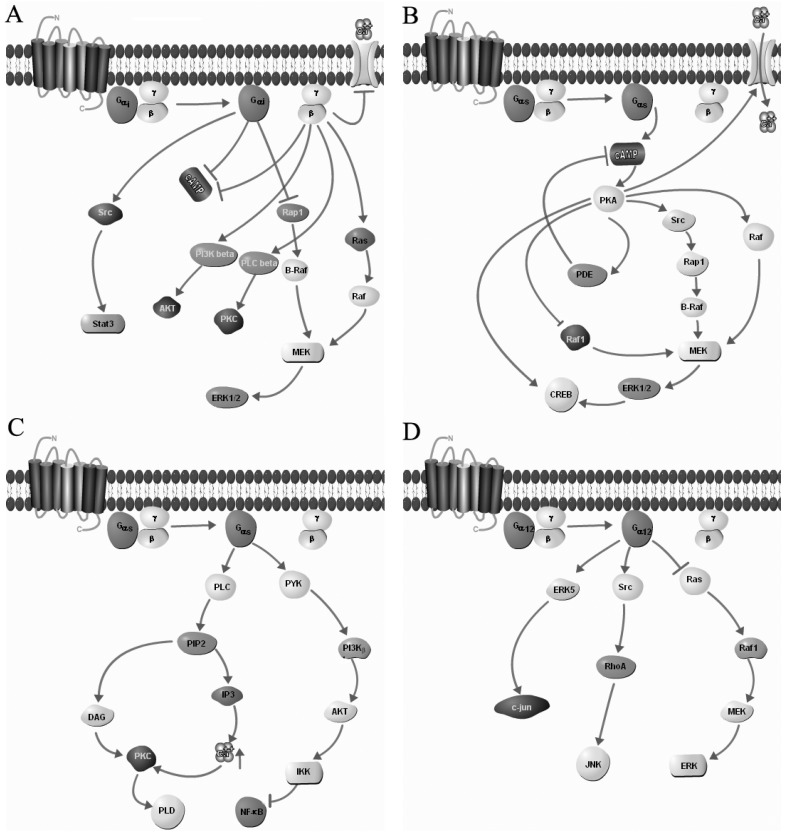
Schematic presentation depicting the regulation of receptor signaling through G-proteins. GPCRs upon activation couples to G_αs_ (**A**), G_αi_ (**B**), G_αq_ (**C**) or G_α12_ (**D**) and regulates signaling in specific manner. Downstream signaling involving G_βγ_ subunit is depicted in panel A [78,79,81]. Figure was made by using the online pathway builder from Protein Lounge [126].

At large, G_s_ is related with the activation of AC isoforms 1–9. Functionally, increased production of cAMP as the second messenger via activation of AC is associated with the GPCR’s signaling via G_s_ protein (Figure 1A) [79]. G_i_ inhibits AC isoforms 5 and 6. Physiological response of many hormones and neurotransmitters like dopamine, epinephrine, and somatostatin (SST) is via G_i_ protein. Members of the G_i_ family are pertussis toxin (PTX) sensitive as it blocks the receptor coupling and downstream signaling by catalyzing ADP ribosylation of position 4 cysteine residue in the α subunit of *C*-terminal (Figure 1B). G_q_ activates PLCβ1-4 and this pathway is activated by calcium-mobilizing hormones which in turn leads to the activation of PLC that activate intracellular messengers inositol trisphosphate (IP3) and diacylglycerol (DAG) [79]. IP3 is involved in calcium release whereas DAG is associated with protein kinase C (PKC) recruitment (Figure 1C). In contrast, G_12_ is involved with the Rho-guanine nucleotide exchange factor (Figure 1D). On the other hand, there are many receptors which have shown multiple G protein coupling. Recent studies have shown association of single GPCR with multiple G proteins like G_i/s_, G_q/s_, G_i/q_, G_q/i/s_ proteins. The coupling of GPCRs to different G-proteins is the physiological manifestation and pharmacological characterization of the diversity in the receptor signaling in cell and tissue dependent manner. The association of receptors to distinct G protein is responsible for the activation of specific effector molecule and stimulation of diverse signaling cascades responsible for varied biological effects by a single receptor. β-ARs, generally believed to couple G_s_ may also bind to G_i_ for signaling under the influence of protein kinase A (PKA) [108,109]. Upon agonist activation, β-ARs couple to AC in G_s_ dependent manner which subsequently increases intracellular cAMP levels. Conversely, β-ARs when coupled to G_i_, activate distinct signaling cascades through the inhibition of cAMP [119,120,121]. Coupling to multiple G proteins is not limited to the β-ARs, other receptors like ORs which couples to G_i_ have also shown coupling with G_s_[122]. In general, SST exert inhibitory effect on AC however, studies have also described dual effect of SST in dose dependent manner in somatotrophs of porcine origin with increased cAMP at low and high concentration [123]. Although receptor subtype associated with such dual effect of SST is not known however SST coupling to different G protein has been speculated [124]. The functional impacts of different G proteins on homeostasis, embryonic and gonadal development as well as in learning and memory have been described elsewhere in details [79,125]. Importantly, it is worth of investigation to elucidate the role of different G proteins in SSTRs heterodimerization, modulation of receptor mediated signalling and its relevance to pathological conditions.

## 3. Homo- and Heterodimerization of GPCRs

Fraser and Venter, using immuno-affinity chromatography and Western blot analysis, put forth direct evidence for the first time for β_2_AR dimerization in cells of lung origin [127]. Similarly, Paglin and Jamieson came forward with their observations on covalent cross-linking of angiotensin receptor 2 to its binding sites in rat adrenal membrane [128]. Furthermore, number of other receptors were identified in larger entity than expected monomeric size by using radiation inactivation and photo-affinity labeling experiments [129].

The physical evidence for GPCRs dimerization were further supported with studies involving GABA receptors, where receptor functionality and sorting to the cell surface was dependent upon dimerization with other subtypes [130]. The ER retention signal of the GABA_B_R1 was masked upon heterodimerization with GABA_B_R2 leading to proper sorting of the receptors to the cell surface [131]. Further, interaction of both the receptors subtypes was critical for the signaling specifically GABA_B_R1 for the ligand binding and GABA_B_R2 for the G-protein coupling. Cells expressing taste receptors (TRs)-T1R1, T1R2 or T1R3, were insensitive to taste stimuli, whereas heterodimerization of T1R1 with T1R2 and T1R3 was shown to be required for sensation of sweet and umami taste [132]. Although the formation of functional receptor complex upon heterodimerization of two non-functional receptors was often described, dimerization of two functional receptors was shown for the first time for δ and κ ORs. This interaction resulted in distinct ligand binding efficiency of the receptor complex [133]. Homo- and heterodimerization of ORs imparted distinct pharmacological and functional properties to the receptor in comparison to its monomers [50,70,134]. Many other unrelated studies also suggested dimerization as the basic requirement for GPCRs signaling. Interestingly, studies have also shown receptor monomers as the functional units for example SSTR1 as discussed in details at later point in this review [21].

Radioligand binding studies indicated that SSTR2 is the most prominent receptor subtype expressed in the murine central nervous system (CNS). SSTR2 knock out (*ko*) animals displayed significant loss in SST binding in comparison to other SSTR *ko* animals [135]. Furthermore, the changes in the pharmacological properties of SSTR subtypes upon homo- and/or heterodimerization might also be responsible for the loss of SST binding in *ko* animals [136,137]. In contrast to our previous studies in SST *ko* mice, no compensatory changes in other SSTRs expression were observed in SSTR2 *ko* mice [135,138]. On a similar note, distinct pharmacological properties of SSTRs observed in cells and neoplastic tissues indicate the existence of additional undiscovered SSTR subtypes. However, SSTRs interaction leading to the formation of homo- and/or heterodimers might be the possible explanation for the distinct pharmacological properties [137,139,140,141]. By using a combination of morphological, pharmacological, biochemical and biophysical techniques, there are strong evidences for dimerization of SSTRs in agonist dependent manner [15,17,21,30,31,34,35,37,40,52,53,63,64]. The concept of SSTR dimerization was first demonstrated for the SSTR5 subtype using Photobleaching-Fluorescence resonance energy transfer (Pb-FRET) analyses [21]. SSTR subtypes homo- and heterodimerization have also been demonstrated in live cells by Fluorescence correlation spectroscopy (FCS) technique [142]. Later, using Pb-FRET analysis it was demonstrated that SSTR2, SSTR3 and SSTR4 exist as preformed dimers and respond distinctly to agonist treatment [32,36,38]. SSTR1 is the only receptor subtype which exists as monomer irrespective of agonist induced activation [21]. A most interesting result for homodimerization was observed for SSTR2 in different species (human, rat or porcine), which exists as constitutive homodimer in the basal condition and dissociates into monomers upon agonist treatment [32,52,63]. These studies cumulatively suggest that dissociation of SSTR2 in all species is a prerequisite for receptor internalization. Interestingly, elegant observation in porcine SSTR2 demonstrated reassociation of the receptors as dimers immediately after dissociation [63].

Furthermore, in addition to homodimerization, SSTRs also exhibit heterodimerization within the family. SSTR1/5, SSTR2/5, SSTR4/5 and SSTR2/3 display heterodimerization whereas SSTR1/4 does not form heterodimers in basal or upon agonist treatment [21,31,34,36,52]. hSSTR2/hSSTR3 heterodimerization was induced by SSTR2 selective agonist only whereas endogenous pan-agonist SST-14 has shown to enhance hSSTR1/hSSTR5 heterodimers formation [21,31,34,142]. SSTR2 and SSTR3 of rodent origin displayed SSTR2 like characteristics upon heterodimerization, but also exhibit relatively greater resistance to agonist-induced desensitization [52]. SSTR2-mediated SSTR3 inactivation upon heterodimerization was also observed in the cerebellum of developing rat brain that exhibit high levels of SSTR2 and SSTR3 mRNA expression whereas display absence of SSTR3 binding sites [143]. SSTRs are also shown to form heterodimers with receptors of other members of GPCR family. Previous studies from authors lab have demonstrated heterodimerization of D2R with SSTR2 and SSTR5 and its regulation by agonist-binding [30,35]. Heterodimerization between SSTR2 and the μ-opioid receptor did not altered the ligand binding properties of receptor but modulates the receptors phosphorylation, desensitization and internalization [53]. Recently, we have also shown the receptor and ligand dependent heterodimerization of β-ARs with SSTR5 in regulation of receptor expression and downstream signaling in heterologous system [37,40].

Later, another insight for GPCRs homo/heterodimerization was introduced from studies demonstrating ligand-induced regulation of GPCR dimerization. For the members of Class A GPCR subfamilies, co-activation of the receptors in the dimer pair has been shown as a prerequisite for the stabilization of heterodimers [62,72,144,145,146,147], whereas activation of one interacting receptor protomers was also found to equally foster heteromeric interactions [30,33,34,35,148]. Like CXCR4 (chemokine receptor 4), SSTR5 exist in monomeric state and homodimerization is enhanced upon activation with their respective ligands [34,149]. Constitutive SSTR4 dimers display an enhanced dimerization upon ligand activation whereas ligand binding induces a reverse effect on SSTR3 homodimers [36,38]. In contrast, dissociation of preformed human thyrotropin receptor and Neuropeptide Y4 receptor homodimers was induced upon agonist activation [150,151]. Ligand binding is not always critical regulator of receptor dimerization, some of the receptors have been shown insensitive to ligand such as melatonin receptor 1 (MTR1) and the murine SSTR2 and SSTR3 heterodimers [52,152]. Recently, we have shown that SSTR5 and βAR heterodimerization reduces upon agonist treatment whereas combined agonist treatment fosters this interaction [37]. In contrast, dissociation of receptor homodimers was also suggested essential for proper receptor trafficking. For instance, dissociation of δOR homodimers promotes down regulation of the receptors at the membrane and blocking this dissociation by using cross-linking reagents resulted in the impaired internalization process [134]. Similar observations have also been made for SSTR2, as ligand activation induces dissociation of receptor homodimers and receptor internalization [32]. However, in contrast internalization was increased in case of the platelet activating factor receptor and the thyrotropin-releasing hormone receptor upon dimerization [153,154].

## 4. Implications of Structural Domains in GPCR Dimerization

Several previous studies have revealed the functional significance and involvement of different structural domains of GPCRs in the process of homo- and/or heterodimerization. Apart from the critical role played by the different structural domains of GPCR in receptor dimerization, these structural domains are also crucial for eliciting physiological, functional and pharmacological properties of the receptors. Many structural interfaces have been anticipated as critical contributors for the GPCRs dimerization including the amino terminal domain, intracellular loops, transmembrane domains and *C*-terminal. *N*-terminal of GPCRs is of variable size in different receptors and plays critical role in protein folding, intracellular trafficking and ligand binding. For GABA receptors, *N*-terminal of the GABA_B_R1 and GABA_B_R2 subunits are essential for the coupling of GABA_B_ receptors to G_i_ and G protein activated K channels (GIRKs) [155]. Similarly, *N*-terminal variants of serotonin receptor (5-HT) and µOR have shown altered ligand binding in comparison to the wild types whereas *N*-terminal variants of β_2_AR displayed changes in the agonist mediated receptor internalization [156,157,158]. 5-HT receptor also display modulated receptor trafficking whereas no distinct change in ligand binding was observed [159]. The extracellular amino-terminal domain has been shown to be critical for the glutamate receptor and CaSRs dimerization [160,161] as well as for the bradykinin 2 receptor (B2R) homodimerization [162]. Dimerization of glutamate and CaSR is via disulphide linkage between cysteine residues of *N*-terminal which is significantly altered upon mutations in Cysteine residues of *N*-terminal [160,163,164].

*C*-terminal of GPCRs act as a potential site for G-protein receptor kinase (GRKs) mediated phosphorylation, desensitization as well as palmitoylation. The *C*-terminal has been associated in the dimerization for the GABA_B_Rs and αOR [56,134]. The GABA_B_R dimerize via the cytoplasmic tail (*C*-tail) in the endoplasmic reticulum a process independent of ligand activation [56,57,165]. SSTR subtypes present a very unique scenario where each member behaves in a different way for homo- and/or heterodimerization. Importantly, *C*-tail is critical determinant of SSTR dimerization in receptor specific manner. The *C*-tail is markedly not a prerequisite in case of SSTR3 homodimers whereas, SSTR4 losses the capability of homo- and heterodimerization with the deletion of *C*-tail [36,38]. Switching SSTR4 *C*-tail with SSTR1 resulted in a chimeric SSTR4 which displays impaired surface expression and dimerization. Interestingly, in the presence of the *C*-tail of SSTR5, SSTR4 retains the capability of homodimerization [36]. While these studies delineate *C*-tail as a crucial factor in SSTRs oligomerization specifically negative role of SSTR1 *C*-tail in dimerization, future studies will be of pharmacological significance once the composition of amino acid in *C*-tail of SSTR1 responsible for this effect is determined.

Transmembrane domains embedded in the membrane are α-helical structures. Variation in α-helices of D4R, follicle-stimulating hormone receptor (FSHR) and vasopressin receptor 2 (V2R) resulted in attenuated ligand binding, altered coupling to adenylyl cyclase and defective receptor trafficking respectively [166,167,168]. Intracellular loops (ICLs) are implicated in receptor signaling as well as for interactions with regulatory proteins like arrestins or GRKs. Mutations in ICL of D2R, endothelin receptor (ETbR) and V2R alters receptor coupling to G proteins [166]. The TMs, extracellular loop (ECL) and ICLs for several GPCRs are implicated in the process of dimerization including rhodopsin receptors, DRs (ICL III), β-ARs (TMD VIth) and α_1b_-adrenoreceptor [28,30,169,170]. Additionally, homodimerization of CXCR2 involves multiple regions including ECL1, TM III, and ICL II [51].

## 5. GPCR Trafficking and Ligand Binding Is Altered upon Dimerization

Functional diversification of receptors dimerization has been observed in ligand binding, trafficking and signal transductions. Depending upon the receptor status, ligands have distinct affinity and accessibility to the binding sites. For instance, enhanced ligand binding for purinergic receptor (P2Y1) and decreased ligand binding for α-AR has been shown following the formation of heterodimers [171]. There are many such evidences to support the fact that one of the receptor in dimer pair modulates the properties of the partner receptor. For instance, muscarinic receptor (MR) subtypes M2R and M3R have distinct pharmacological properties upon heterodimerization in comparison to its monomers [42]. β_2_AR mediated airway smooth muscle relaxation is reduced upon heterodimerization with prostaglandin E1 receptor (EP1) whereas the β_2_AR displayed enhanced affinity for the ligand upon heterodimerization with β_1_AR [148]. Similar changes in the ligand binding affinity of µ, δ, and κ receptors agonist were observed upon δOR and κOR/µOR heterodimerization [49,133]. Heterodimers of µOR and δOR were shown to be PTX insensitive whereas the monomers and homodimers were sensitive [49].

Heterodimerization may provide negative or positive cooperativity to the ligand binding. SSTR2 and SSTR3 forms constitutive heterodimers and display significant reductions in binding affinity for SSTR3-selective agonist L-796,778 [52]. Furthermore, SSTR3/2 heteromeric complex exhibit SSTR2 like properties in GTP binding, inhibition of AC, and phosphorylation of extracellular regulated kinases (ERK1/2). In case of D2R/SSTR2 heterodimers, positive cooperativity was observed for D2R, as the agonist-bound SSTR2 significantly enhanced binding affinity of D2R [30,35]. In addition, simultaneous activation of two interacting protomers enhanced downstream signaling efficiency. Moreover, the modulation of endocytic properties of bradykinin B2 and angiotensin AT1 receptors have also been observed upon heterodimerization [172]. Studies have demonstrated selective preferences of certain docking proteins in receptor internalization in homo- and/or heteromeric complexes. Homodimers were shown to internalize in a clathrin and dynamin dependent manner whereas heterodimers endocytosed in a dynamin dependent manner only [172]. These receptor pairs also display switching in the coupling to G-proteins from G_q_ to G_i_ upon heterodimerization when compared with homodimers respectively. β_1_AR upon heterodimerization with β_2_AR resulted in internalization resistant complex, where the internalization property of β_2_AR was compromised in the presence of internalization resistant β_1_AR [68].

## 6. Implication of GPCR Heterodimers in Pathophysiological conditions

The concept of GPCRs heterodimerization and its association with many pathological conditions is now well appreciated. Several studies have shown that GPCRs in a heterodimeric complex elicit a significant role in a number of diseases at different stages, either via regulating the pathological condition or towards its progression by modulating selective downstream signaling cascades. Some of the pathological conditions associated with receptor dimerization are discussed briefly.

*Acquired immune deficiency syndrome*: Human immunodeficiency virus (HIV) at different stages of infection uses chemokine receptors as co-receptors. Interaction of a mutant for chemokine receptor 2 (CCR2) with CCR5 or C-X-C chemokine receptor type 4 (CXCR4) result in a blockade or delay of HIV infection due to the inability of the virus to bind these later receptors [173]. The heterodimerization between CCR2 mutant CCR2V64I with CXCR4 may result in decreased levels of CXCR4 in peripheral blood mononuclear cells resulting in the delayed progression of the disease [173,174].

*Asthma*: Heterodimers of EP1/β_2_AR receptor plays a critical role in progression of asthma [148]. EP1 receptor in monomeric form has no physiological significance whereas upon heterodimerization with β_2_AR results in altered β_2_AR conformation and decreased signaling. Heterodimerization resulted in modulation of coupling between Gαs and β_2_AR in airway smooth muscles causing reduced bronchodialatory potential of agonist [148]. The activation of EP1 receptors increases down-regulation of heterodimer pair, resulting in loss of β_2_AR binding to G_s_ which diminishes β_2_AR-mediated airway smooth muscle relaxation significantly [148].

*Cardiac Failure*: Blockade of AT_1_R and β-ARs signaling has been shown beneficial in cardiac failure. β-ARs antagonist via trans-inhibition blocks Ang II mediated pathways thus regulating two different receptors mediated signaling involved in the pathophysiology of the failing heart. Studies have also demonstrated that catecholamine mediated heart rate is regulated by AT1R and β_2_AR heterodimers [175], whereas the regulation of cardiac contractility by AT1R/β_2_AR as well as β_1_AR/β_2_AR heterodimers has been shown which regulates desensitization of β_2_ARs upon agonist activation [176].

*Preeclampsia*: Several previous studies have suggested the role of AT_1_R/B2R heterodimers in conditions like hypertension, preeclampsia and smooth muscle cells contraction [176,177,178]. Studies have also suggested increased expression levels of AT_1_R/B2R heterodimers in renal mesengial cells of hypertensive rats in comparison to normal rats. Upregulation of AT_1_R/B2R heterodimers during pregnancy plays critical role in AT_1_R mediated hyper-responsiveness in hypertension (in preeclampsia) [177,179,180] whereas Ang II mediated signaling was negatively regulated by selective inhibition of heterodimer pair [180].

*Psychosis/Schizophrenia*: Modulation of the 5-HT and glutamate receptor functional complex has been shown to play critical role in inclining schizophrenic patients to psychosis [181]. In untreated schizophrenia patients, increased level of the 5-HT and decreased level of glutamate receptor was observed in the post-mortem brain. Recent studies have proposed that drugs used in schizophrenia may be targeting different receptors in the serotonin/glutamate receptor heterodimer pair thus playing critical role in triggering distinct downstream cellular pathways [182].

*Parkinson’s Disease (PD)*: Heterodimerization of A_2a_R and D2R has been exploited as a potential target in improving the side effects of L-DOPA in PD [183]. Dyskinesia (uncontrolled muscle contractions) was major issue with administration of L-DOPA for the treatment of PD initially [184]*.* A_2a_R agonist modulates cell surface expression of D2R resulting in decreased binding affinity for D2R agonist [185,186] whereas the use of A_2a_R antagonist improves motor control without causing dyskinesia in mouse model of PD [187]. Neurodegenerative diseases hold better potential for the development of new therapeutic drugs due to the widespread distribution and complex communicating network of several members of GPCR, ionotropic glutamate receptors and RTK family in CNS.

## 7. Implication of Somatostatin Receptors Heterodimerization in Pathological Conditions

Five SSTR subtypes in addition to have a common affluence to inhibit AC have shown unique characteristic for the formation of homo- and heterodimers with significant diversification and represents a promising target in several pathological conditions like cancer, pain, heart failure and neurological disorders [188,189].

Several previous studies have shown the expression of one or more SSTRs including SSTR2 and SSTR5 in tumor tissues of different origins like breast, prostate, pituitary and pancreas [190,191,192,193]. Many studies have also shown the co-expression of SSTRs and DRs in tumor of different origins [194]. As discussed in previous section, SSTR2 and 5 heterodimerize with D2R *in vivo* and *in vitro* experimental conditions and this association has emerged as the potential target in the treatment of pituitary adenomas [30,35,192]. Chimeric molecules targeting two receptors or three receptors have been developed and used successfully to regulate the growth hormone and prolactin secretions *in vitro*. These chimeric molecules (dopastatins) were developed to target SSTR2, SSTR5 and D2R as a potential target for the treatment of pituitary tumor “acromegaly” [195,196,197,198,199]. Several previous studies have demonstrated resistant to octreotide therapy in patients with growth hormone secreting tumors [200,201]. Recently, Castano’s group suggested that SSTR mutants might result in the non-responsiveness of these patients to SST analogues therapies [64,202,203]. Mutant of SSTR5 like SST5TMD4 is expressed in normal as well as in breast tumor tissues along with other wt SSTR subtypes [204]. Moreover, the heterodimerization of SSTR2 with mutated SST5TMD4 modulates SSTR2 regulated downstream signaling and SST mediated anti-tumor effects [204].

Studies have shown that all five SSTR subtypes are variably expressed at the mRNA and protein levels in breast tumor cells [205,206,207,208,209,210,211,212]. Interestingly, SSTR expression is positively correlated with tumor size, whereas inversely correlated with EGFR expression levels and tumor differentiation [210,211]. In a number of *in vitro* and *in vivo* mammary cancer models, SST and its analogs displayed anti-tumor activity for, e.g., OCT decreases the growth of ER^+^ cell lines in culture [213,214,215,216,217,218,219,220]. Recent studies have shown that SSTRs specifically SSTR1 and SSTR5 alters ErbB1 signaling and abrogate EGF-stimulated cell proliferation and signaling [15,16,17]. These observations are of significance in the pathophysiology of tumors, such as breast cancer. SSTRs interfere with the homo- and heterodimers of ErbBs and modulate the downstream signaling significantly via activating different signaling molecules thus promoting cytostatic or cytotoxic effects rather than proliferation [15,16,17]. The specific function of SSTRs in breast tumor is still not clear, but can be exploited for diagnostic and therapeutic approaches. Employing SST and its analogues in combination with chemotherapeutic agents might provide an additional tool to target tumours in multiple ways or to provide synergistic anti-tumor actions.

Like SST, the opioid transmitter system is also widely expressed throughout the brain and exert crucial role in pain perception, consciousness, motor control and autonomic function. These effects are mediated through ORs that comprise three subtypes-µ, δ, and κ, that are structurally related to SSTRs and share ~40% sequence homology. Recent studies have demonstrated that SSTRs also play a critical role in mediating analgesic effects of SST [188,221,222]. For instance, upon direct administration to the central and peripheral nervous system in animal model of post-operative and neoplastic pain, the SST analogue octreotide is both analgesic and morphine-sparing [223,224]. Octreotide has been observed to behave as an antagonist in morphine-dependent individuals and patients undergoing morphine withdrawal have presented with reduced vomiting following octreotide administration [225,226]. Recent studies have shown SSTR4 and δORs colocalize in rat brain and spinal cord and also exhibit heterodimerization in heterologous system [39]. Pfeiffer *et al*., demonstrated that SSTR2 and the μOR co-localized in neurons of the locus coeruleus and SSTR2 with µOR exhibit heterodimerization in transfected cells, without any significant changes in the receptor pharmacology and signaling [53]. At present, opioids are the best available options as analgesic drugs in the treatment of pain, whereas there are several side effects associated with the prolonged use of opioids, including addiction and withdrawal. We propose that SST analogs in combination with opioid receptor agonists will minimize the side effects of opioids and thus will serve as potential safer therapeutic agent for pain relief in future.

The role of β-ARs in heart failure and cardiac complication is undisputed. Recent studies have also described that SST play an important role in regulation of cardiac contraction. There is other evidence supporting the fact that 30–40% deaths in Huntington’s disease and pituitary tumor (acromegaly) are due to heart failure [227,228]. Whether the gradual loss of SST, uncontrolled GH and IGF-1 secretion, or the modulation of cellular functions are responsible for heart failure is not yet clear. Importantly, these studies support intimate association between SST and heart failure. Recent studies have shown direct evidence that SSTR subtypes functionally interact with β-ARs [37,40]. Previous studies as early as in 1985 pointed out this association and provided the evidence that β-AR mediated cAMP was enhanced in presence of SST in astrocytes prepared from rat brain without displaying any changes in β-AR binding properties [229]. Interestingly, in cultured astrocytes SST alone has no effect on cAMP. SST has been shown to promote the membrane translocation of β-AR kinase similar to the effect of isoproterenol. The presence of cytosolic enzyme β-AR kinase is a prerequisite for β-AR phosphorylation in presence of agonist [230]. Recent studies from HEK-293 cells stably transfected with SSTR5 and β-ARs have shown the existence of basal heterodimers strengthen the concept of functional interaction between adrenergic and somatostatinergic system with possible role in cardiac tissue [37,40,231]. Future studies involving SSTR and β-AR subtypes will uncover the role of SSTR and AR subtypes in the cardiovascular system. Most importantly, studies directed in experimental condition when β-ARs switch their coupling from G_s_ to G_i_ will be of particular interest in combination with SSTR subtypes.

## 8. Conclusions

At the time that this review was written, it was well established and largely accepted that GPCRs function in an integrated manner as homodimers and/or heterooligomers and serve as potential platform for the development of new therapeutic approaches in certain pathological conditions. There are many other members of GPCR family which have not been explored for their exact physiological functions in a heteromeric complex yet. The development of new methodology to elucidate the distributional pattern and receptor orientation in heteromeric complex at cell surface will help in delineating the protein-protein interaction and physiological significance of such complex formation. However, additional studies are warranted specifically in a system where these receptors are expressed endogenously like in CNS, often expressing more than one receptor in a single cell. Considering these examples as described in this review amplifies the concept that it is the heteromeric complexes and not monomers which are playing critical role in regulating most if not all pathophysiological conditions. The intensity of heterodimers is sometimes elevated in diseased conditions and only thus becomes an easy target for drugs. Modulation of receptor functions, signaling and more significantly the expression level has been proven critical upon heterodimerization and compounds regulating these functions have proved beneficial in many diseases including pituitary adenomas. Taken in consideration drugs targeting specifically heteromeric complex would be new therapeutic interventions in the time to come.
